# Cantilever-enhanced dual-comb photoacoustic spectroscopy

**DOI:** 10.1016/j.pacs.2024.100605

**Published:** 2024-03-21

**Authors:** Jiapeng Wang, Hongpeng Wu, Xiaoli Liu, Gang Wang, Yong Wang, Chaofan Feng, Ruyue Cui, Zhenfeng Gong, Lei Dong

**Affiliations:** a State Key Laboratory of Quantum Optics and Quantum Optics Devices, Institute of Laser Spectroscopy, Shanxi University, Taiyuan 030006, China; b Collaborative Innovation Center of Extreme Optics, Shanxi University, Taiyuan 030006, China; c Laboratoire de Physicochimie de l′Atmosphère, Université du Littoral Côte d′Opale, Dunkerque 59140, France; d School of Optoelectronic Esssngineering and Instrumentation Science, Dalian University of Technology, Dalian, Liaoning 116024, China

**Keywords:** Photoacoustic spectroscopy, Dual-comb spectroscopy, Optical cantilever, Gas sensing

## Abstract

Dual-comb photoacoustic spectroscopy (DC-PAS) advances spectral measurements by offering high-sensitivity and compact size in a wavelength-independent manner. Here, we present a novel cantilever-enhanced DC-PAS scheme, employing a high-sensitivity fiber-optic acoustic sensor based on an optical cantilever and a non-resonant photoacoustic cell (PAC) featuring a flat-response characteristic. The dual comb is down-converted to the audio frequency range, and the resulting multiheterodyne sound waves from the photoacoustic effect, are mapped into the response frequency region of the optical cantilever microphone. This cantilever-enhanced DC-PAS method provides advantages such as high sensitivity, compact design, and immunity to electromagnetic interference. Through 10 seconds averaging time, the proposed approach experimentally achieved a minimum detection limit of 860 ppb for acetylene. This technology presents outstanding opportunities for highly sensitive detection of trace gases in a wavelength-independent manner, all within a compact volume.

## 1 Introduction

Optical frequency combs (OFC) [1], [2], [3] provide equally spaced spectral lines from ultraviolet to infrared [4], [5], and can be used to link an unknown optical frequency to a radio or microwave frequency reference. Since their inception, OFCs have led a revolution in frequency metrology and precision measurements [6], [7]. Due to its broad spectral coverage and high resolution, interest in OFC has spawned a number of new methods, technologies and applications for spectral analysis such as broadband laser-based gas sensing [8], [9], [10] and molecular fingerprinting [11]. Among these, dual-comb spectroscopy (DCS), based on two OFCs with a slight difference in their repetition frequencies, has emerged as a particularly attractive technique due to its numerous advantages, including high resolution, wide spectral coverage and fast data acquisition [9], [12], [13]. DCS achieves spectral analysis by converting the optical frequencies to radio frequencies through beat frequency and detecting by a photodetector (PD). The absorption information of each beat-note component can be obtained by Fourier-transforming the output of the PD.

DCS offers remarkable capabilities, however, it also presents challenges, including the need for wide spectral response detectors and the employment of high-power OFC without attenuation. Specifically, the ultra-broadband dual combs necessitates a wavelength-independent detector capable of responding to the entire spectral range without changing PDs for different spectral ranges [14], [15], especially for the longer infrared [16]. Additionally, many infrared or visible comb sources can easily generate optical power in the range of hundreds of milliwatts, even in mid-infrared [17], [18], potentially leading the PD into nonlinearity or full saturation if not attenuation. Furthermore, the dynamic ranges of PDs can vary, but for instance, a commercial 100 MHz InGaAs amplified photoreceiver may exhibit a limited dynamic range of approximately 500. This is determined by the ratio of the maximum peak power to the noise-equivalent power integrated over a bandwidth. Consequently, the signal-to-noise ratio (SNR) is inevitably limited by the PD’s dynamic range [12]. Electro-optic sampling [19], [20] can effectively address the performance shortcomings of long-infrared PDs and offer excellent sensitivity and speed; however, it should be noted that it requires precise optical adjustments and has a bulky size.

These challenges have become increasingly significant as dual-comb sources continue to evolve. To address them, researchers have explored the integration of DCS with photoacoustic spectroscopy (PAS), resulting in a novel technique known as dual-comb photoacoustic spectroscopy (DC-PAS) [14], [21], [22], [23]. PAS is a powerful analytical technique that enables the quantification of trace gases through acoustic detection of their indirect absorption information [24], [25], [26]. In PAS, the absorbed light energy creates heat in the sample, causing thermal expansion and ultimately generating a pressure wave or sound wave. Since sound waves are measured in PAS instead of light waves, the wavelength-dependent PDs in DCS is eliminated. Conventional DC-PAS systems often utilize an acoustic-electric transducer such as a MEMS microphone and a condenser microphone as the detector to capture the photoacoustic signals, due to their high sensitivity and excellent stability. In this case, wavelength-independent and compact size DCS can be realized at the expense of certain data acquisition speed and optical bandwidth compared with typical DCS, due to the limitation of molecular relaxation rate and acoustic detection bandwidth.

For acoustic sensing, sensitivity is fundamentally limited only by the random momentum kicks from gas molecules as they collide with the sensor when the acoustic wave propagates through the gas [27], [28]. However, typical acoustic-electric transducers are far from reaching this limit due to electronic or thermal noise. In the traditional DC-PAS, this issue is also evident because the PA signal is detected by a typical acoustic-electric transducer, such as electret condenser microphones or quartz tuning forks [26], [29], [30], [31]. The situation becomes worse when there is significant electromagnetic interference or when remote measurements are performed.

Optical cantilever microphones rely on the interference of light waves to measure minute displacements induced by sound waves [32], [33], [34], [35], [36], [37], [38], [39]. Such all-optical detection provides acoustic detection with ultra-high sensitivity. Therefore, when using an optical cantilever microphone for DC-PAS detection, there is potential to achieve a higher signal-to-noise ratio (SNR). Furthermore, a non-resonant PAC with a closed cavity structure can be used to confine the sound waves generated by the photoacoustic effect and further enhance the detection sensitivity when working together with the optical cantilever microphone.

In this manuscript, we adopted a Fabry-Perot optical cantilever microphone and a non-resonant PAC to complete the cantilever-enhanced DC-PAS detection, in which the multiheterodyne process of two OFCs induces multiheterodyne sound waves through the photoacoustic effect. The frequency component of multiheterodyne sound waves forms an audio frequency comb. The non-resonant PAC provides a flatness response (∼kHz), which makes a uniform acoustic gain for the multiheterodyne sound waves. The multiheterodyne sound waves drive the multimode vibration of the cantilever beam. The sound waves within the response range of the cantilever can be further enhanced. Then a probe beam is used to demodulate the vibration information of each frequency component by measuring the movement of the cantilever using a laser Fabry-Perot interferometer (FPI). The combination of non-resonant PACs and optical cantilever microphones is highly appealing for DC-PAS technology as it can achieve small volumes, high sensitivity, and all-optical detection for DC-PAS.

## 2 Principles

### 2.1 Generation of multiheterodyne sound waves

**Fig. 1:**
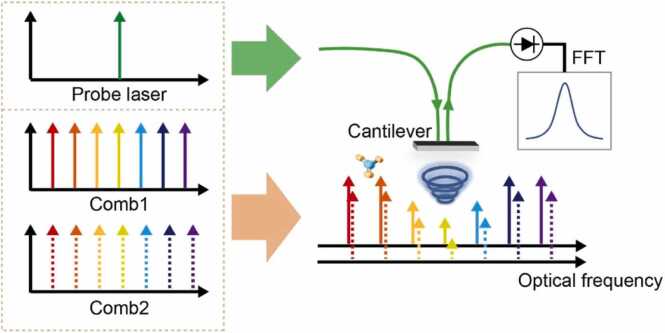
Principle of cantilever-enhanced DC-PAS. Dual-comb interacts with gas molecules and produces multiheterodyne sound waves through the photoacoustic effect. The multiheterodyne sound waves reach the surface of the cantilever beam and cause the multimode vibration of the cantilever beam. A probe beam is irradiated to the surface of the cantilever, and the absorption information of the gas can be inverted by demodulating the intensity changes of the reflected light by FFT.

The cantilever-enhanced DC-PAS concept is illustrated in Fig. 1. An optical cantilever microphone is used to detect the multiheterodyne sound wave generated by the photoacoustic effect due to the periodic relaxation of molecules. In our apparatus, the dual-comb source is generated through electro-optic modulation, due to its remarkable tuning capabilities and high mutual coherence. The optical frequency components $f_{n,1}$ and $f_{n,2}$ of two optical frequency combs can be expressed as

(1) $$f_{n,1}=nf_{\mathrm{rep},1}+f_{0,1}+f_{\text{cw}}$$

(2) $$f_{n,2}=nf_{\mathrm{rep},2}+f_{0,2}+f_{\text{cw}}$$

where $f_{\mathrm{rep},1}$ and $f_{\mathrm{rep},2}$ represent the repetition rates of the two combs, respectively, $f_{\text{cw}}$ is the optical carrier frequency, which is identical for the two combs when they are generated from the same continuous-wave (CW) laser. The optical frequency shift of the combs is determined by $f_{0,1}$ and $f_{0,2}$, in order to ensure the uniqueness of the acoustic frequency after down-conversion. The subscript 1 and 2 identify the two combs, and n=0,±1,±2,... represents the modulation order of electro-optic modulation and the comb line indices. It is assumed that $f_{\text{rep},1}>f_{\text{rep},2}$ and $f_{0,1}\;>\;f_{0,2}$. When two combs are optically combined, this can be interpreted as modulations with frequency $f_{n}$ in optical power.

(3) $$f_{n}=f_{n,1}-f_{n,2}=n(f_{\mathrm{rep},1}-f_{\mathrm{rep},2})+f_{0,1}-f_{0,2}$$

(4) $$=n\times \Delta f_{\mathrm{rep}}+f_{0}$$

It is worth noting that the difference in the repetition rates and the optical frequency shift of the two combs is set very small, i.e., $\Delta f_{\mathrm{rep}}\ll f_{\mathrm{rep},1}\approx f_{\mathrm{rep},2}$, $f_{0}\ll f_{0,1}\approx f_{0,2}$. The audio frequency comb is generated by the multiheterodyne process. The dual comb excites the target gas through the photoacoustic effect, producing multiheterodyne sound waves that drive the multimode vibration of the optical cantilever. A probe laser is irradiated to the interface of the cantilever beam, and the multimode vibration information of the cantilever can be obtained by demodulating the intensity changes of the reflected light by FFT, and further the absorption information of each beat-note pair can be obtained.

### 2.2 FPI-based cantilever microphone

**Fig. 2:**
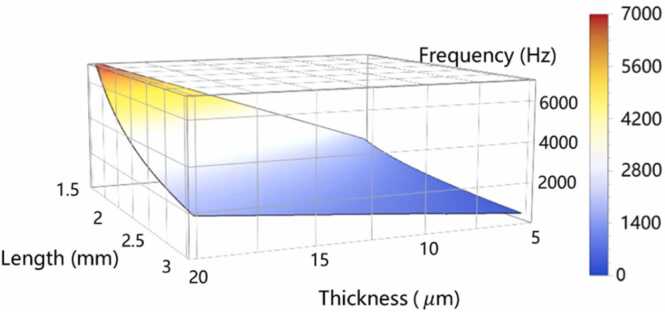
Resonant frequencies of the optical cantilever as functions of length and thickness.

**Fig. 3:**
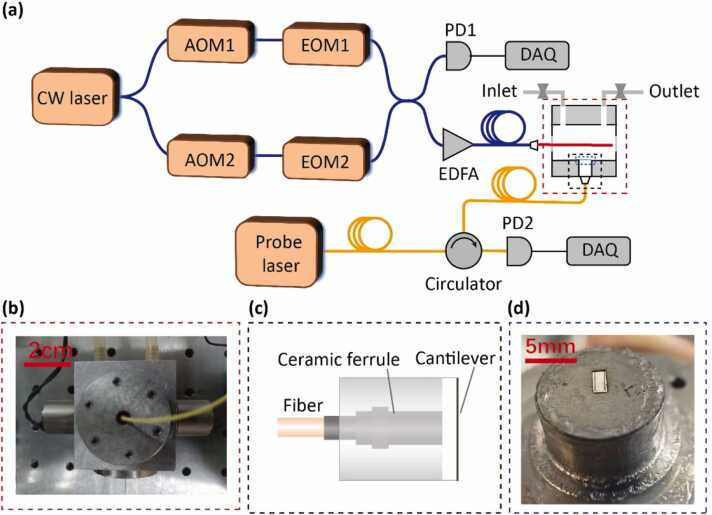
**Schematic of the cantilever-enhanced DC-PAS.** CW laser－continuous wave laser, AOM－acousto-optic modulator, EOM－electro-optic modulator, EDFA－erbium-doped optical fiber amplifier, PD－photodetector, DAQ－data acquisition. **(a)** A dual comb interacts with gas molecules in a non-resonant PAC, the multiheterodyne sound waves push the multimode vibration of the cantilever. A probe laser is irradiated to the interface of the cantilever. The intensity changes of the probe laser are detected by PD2 and then recorded by DAQ. **(b)** Image of the non-resonant PAC. It consists of the fiber interfaces of the CW laser and the probe laser, as well as the inlet and outlet of the gas. **(c)** Schematic structure of FPI cantilever sensor head. The sensor head is composed of a stainless-steel shell, a fiber, a ceramic ferrule and a cantilever. **(d)** Image of the cantilever. The cantilever which is manufactured by laser micromachining technique, has a size of 2 mm × 1 mm and a thickness of 10 μm.

The FPI-based cantilever microphone is specifically designed to detect acoustic waves at low-frequency domains (∼kHz) due to the requirement of molecular relaxation rate. The first-order resonant frequency of a rectangular cantilever can be expressed as [39]:

(5) $$f_{0}=\frac{\tau }{2\pi l^{2}}\sqrt{\frac{2E}{3\times 0.647\rho }}$$

where *E* is Young’s modulus, ρ is the density, τ and *l* are the thickness and the length of the cantilever, respectively. For 304 stainless steel *E* = 194,020 MPa and ρ = 7.93 g/cm^3^. With Eq. (5), the resonant frequency of the cantilever, which is changed with τ and *l*, is shown in Fig. 2,

In order to get the vibration information of the optical cantilever microphone, the reflected light intensity is retrieved by the method of the FPI intensity demodulation. The Fabry-Perot cavity is formed by the air gap between the fiber enface and the cantilever, as shown in Fig. 3(c). When the multiheterodyne sound waves are applied to the surface of the cantilever beam, they cause the length of cavity changed periodically. Due to the low reflectivity of the fiber endface, the FPI can be simplified as a two-beam interferometer. Therefore, the reflection light intensity for different frequency components $I_{r}(n)$ can be described by [40], [41]:

(6) $$I_{r}(n)=2I_{0}[1+\gamma \cos(\frac{4\pi }{\lambda }(d_{0}+\Delta d\sin(f_{n}t))+\pi )]$$

where $I_{0}$ is the intensity of the incident light, γ is the fringe visibility of the FPI sensor, λ is the wavelength of the probe laser, $d_{0}$ is the static cavity length, Δd is the amplitude of the cavity length variation, $f_{n}$ is the frequency of the audio comb for different beat-note pairs, and *t* is the time. Therefore, the acoustic signal can be recorded by the changes in reflected intensity, and demodulated by interferometric intensity demodulation through FFT. For weak acoustic signals, the variation of the reflection intensity can be expressed as:

(7) $$\Delta I_{r}(n)=2I_{0}\gamma \frac{4\pi }{\lambda }\Delta d\sin(f_{n}t)$$

Eq. (7) shows that the reflected intensity will linearly change with the cavity length by the frequency of $f_{n}$. Hence, the multiheterodyne sound waves are converted into the intensity of the reflected light by the PD. The gas absorption information is obtained from the output electrical signals through a direct FFT data processing procedure.

## 3 System and results

The experiment schematic of the cantilever-enhanced fiber-optic DC-PAS apparatus is illustrated in Fig. 3(a). An ultra-narrow linewidth whispering gallery mode laser, emitting around 1531 nm, was selected as the excitation source due to its compatibility with the C_2_H_2_ absorption line and meeting high-resolution requirements. The CW laser is split into two branches, each undergoing frequency shifting by $f_{0,1}$ and $f_{0,2}$ through acousto-optic modulators (AOMs) respectively. The frequency shifts determine the center frequency of the down-converted dual-comb, and ensure the uniqueness of the acoustic comb frequency. Subsequently, each beam is transmitted through an electro-optic modulator (EOM) to generate an OFC with a repetition frequency $f_{\mathrm{rep},1}$ and $f_{\mathrm{rep},2}$ respectively. The dual-comb source is generated by combining two OFCs that after the AOMs and the EOMs respectively. 99% of the combined dual-comb power is introduced into an erbium-doped fiber amplifier (EDFA) for power amplification, reaching 50 mW, before being incident into the non-resonant PAC, which contains the target gas sample. The multiheterodyne sound waves generated in the non-resonant PAC reach the interface of the cantilever and drive its multimode vibration. The images of the non-resonant PAC and the cantilever are shown in Fig. 3(b) and (d). The schematic diagram of the FPI cantilever sensor head, which is made up of a single-mode fiber, a stainless-steel shell, a ceramic ferrule and a stainless-steel cantilever, is shown in Fig. 3(c). A distributed feedback (DFB) laser with a power of 10 mW is irradiated to the interface of the cantilever is utilized as the probe laser to detect the cavity length changes. The reflected light reaches the photodetector (PD2) through a circulator. A fast Fourier transform (FFT) program is used to process the DAQ data from PD2. One percent of the combined dual-comb is monitored by an InGaAs PIN photodetector (PD1), to provide a synchronous reference signal for normalizing the intensity background of the beat-note pairs. Notably, PD1 and PD2 are hardly saturated, because they are not involved in receiving the excitation light.

**Fig. 4:**
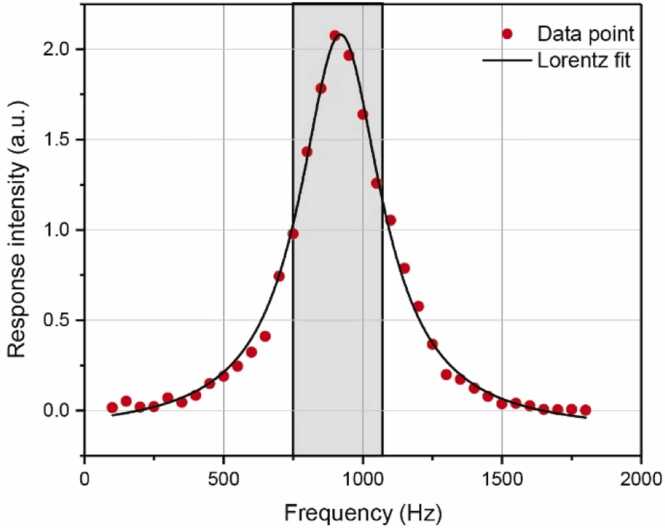
Frequency response of the FPI cantilever microphone. The red points represent the experimental points of the cantilever’s response intensity at different frequency, and the black line shows the result of Lorentz fitting for the experimental points. The gray shaded area represents the detection frequency range chosen.

Fig. 3(b) shows the detection module of the all-optic cantilever-enhanced DC-PAS system, which consists of a FPI cantilever sensor head, a non-resonant PAC, a probe laser, gas inlet and outlet, and electromagnetic valves. The FPI cantilever sensor is consists of a single-mode fiber, a stainless-steel shell, a ceramic ferrule and a cantilever, is shown in Fig. 3(c). The stainless-steel cantilever, shown in Fig. 3(d), has a size of 2 mm × 1 mm and a thickness of 10 μm, corresponding to the resonant frequency around 1 kHz. By frequency scanning from 100 Hz to 1.6 kHz, the frequency response of the FPI cantilever microphone was measured, as shown in Fig. 4.

**Fig. 5:**
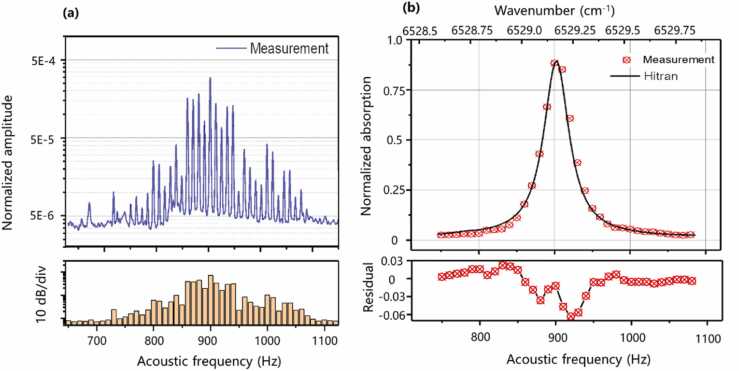
Experimental results of the cantilever-enhanced DC-PAS. **(a)** The cavity length changes at the different acoustic frequencies are retrieved by Fourier transform as shown in the top of Fig. 5(a), and the comb intensity background are given in the bottom of Fig. 5(a), which are used to normalize the measured signal. **(b)** The normalized absorption signal of C_2_H_2_ at 6531 cm^−1^.

We choose acetylene (C_2_H_2_) gas as the sample gas to characterize the cantilever-enhanced DC-PAS system due to the high absorption coefficient of C_2_H_2_ around the 1.5 μm region. Firstly, a 5000 ppmv C_2_H_2_:N_2_ mixture is introduced into the non-resonant PAC, acting as the target gas to detect the C_2_H_2_ P(11) line of the $\nu_{1}+\nu_{3}$ absorption band. When the gas is introduced into the non-resonant PAC, the gas inlet and outlet of the non-resonant PAC are immediately closed by electromagnetic valves. All the measurements are conducted at standard atmospheric pressure and 296 K. The transformed photoacoustic signal is shown in the top of Fig. 5(a), while the comb intensity background is shown in the bottom of Fig. 5(a), which is collected by another photodetector and obtained by performing FFT with the signal simultaneously. The densely packed beatnotes are centered at 900 Hz and have a frequency span from 650 Hz to 1120 Hz and a line spacing of 10 Hz. By normalizing the signal to the comb intensity background and the response curve of the cantilever, the absorption spectrum of C_2_H_2_ is achieved. The frequency resolution of the FFT is set as 1 Hz, which means an acquisition time of 1 s for each spectrum. In this way, the measured absorption features from the average of 10 times are fitted according to the HITRAN database, as depicted in Fig. 5(b). The optical frequency range is from 6528.50 cm^−1^-6529.88 cm^−1^, corresponding to an acoustic frequency range from 750 Hz to 1080 Hz.

### 3.1 Linear response of concentration

**Fig. 6:**
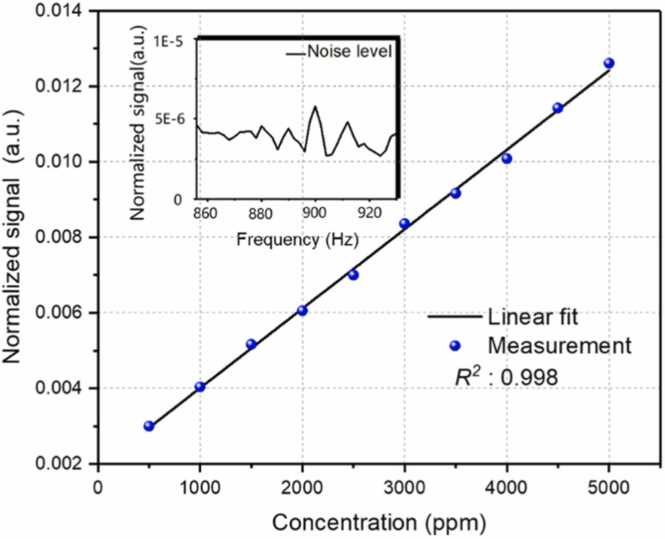
Normalized signal and noise level of the cantilever-enhanced DC-PAS. The blue points show the signal amplitude of the cantilever-enhanced DC-PAS and the insert shows the noise level of the system.

In order to evaluate the linear response capability of the cantilever-enhanced DC-PAS system, different concentrations of C_2_H_2_/N_2_ gas mixtures from 500 ppm to 5000 ppm, generated with the gas dilution system, were introduced into the non-resonant PAC. A dual-comb power of *P* is incident into the non-resonant PAC, where the target gas molecules are effectively excited by the dual-comb source. For the 10 seconds acquisition time, the normalized photoacoustic signal at the absorption line peak, which corresponds to the highest acoustic beatnote (at 900 Hz), as a function of concentration, is presented in Fig. 6. The obtained R^2^ value of 0.998 proves the linear response of the system to the concentration. The insert figure of Fig. 6 shows the typical average level of the noise at the acoustic frequency 900 Hz (corresponding to 5.79E-6), which is obtained by averaging 10 spectra. Therefore, the SNR of the system is ∼581 for the 500-ppm concentration and the minimum detection limit (MDL) is 860 ppb. This value can be further improved by using a longer averaging time (as long as 500 s). Accordingly, the normalized noise equivalent absorption (NNEA) can be calculated by $\frac{P}{N}∙\alpha_{\min}∙\sqrt{t}$. Where N is the number of the spectral lines (*N*=34), $\alpha_{\min}$ is calculated from the HITRAN database, the optical power P is 98 mW and t = 10 s. The side-by-side performance comparison between cantilever-enhanced DC-PAS and other techniques has been shown in Table 1, demonstrating that our technology achieves a comparable NNEA to state-of-the-art technology OFC-PAS in a compact size.

**Table 1:** Side-by-side performance comparison between cantilever-enhanced DC-PAS and other OFC-PAS techniques.

Ref.	Method	Sensing type	Gas	Wavelen-gth (μm)	Time (s)	Detection limit (ppm)		NNEA (cm^−1^·Hz^−1/2^)
[36]	FT-PAS	Cantilever-enhanced	CH_4_	∼3.3	/		0.083	/
[37]	FT-PAS	Cantilever-enhanced	CH_4_	∼3.3	200		0.8	8 × 10^−10^
[14]	DC-PAS	Microphone	C_2_H_2_	1.53	1000		10	/
[9]	DC-PTS	Photothermal	C_2_H_2_	1.53	1000		8.7	/
[23]	DC-PAS	Quartz-enhanced	C_2_H_2_	1.53	100		0.0083	7 × 10^−10^
This work	DC-PAS	Cantilever-enhanced	C_2_H_2_	1.53	10		0.86	8.93 × 10^−9^

### 3.2 Linear response of power

For linear photoacoustic detection, power is a critical parameter, as the signal amplitude is directly proportional to the optical power *P*. To improve the amplitude of the photoacoustic signal, a straightforward way is to increase the optical power. Nevertheless, as the dual-comb power increases, saturation may occur, which implies that the depletion from the vibrational excited level slows with respect to the pump rate and no more molecules are able to be excited to higher energy levels with increasing laser power. In this situation, the PAS signal will not benefit from further higher laser excitation power [42], [43]. In order to avoid the nonlinear response of the system from the complete saturation condition, an experiment to check the saturation level was carried out. A certified gas mixture of 5000-ppmv C_2_H_2_/N_2_ was filled into the non-resonant PAC at standard atmospheric pressure and 296 K.

**Fig. 7:**
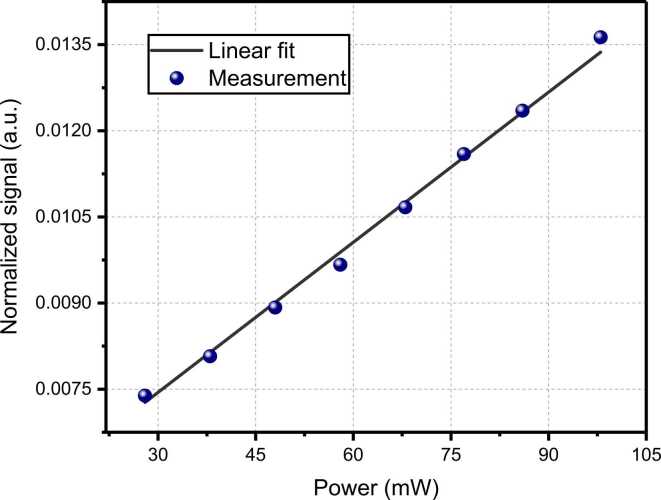
Normalized signal amplitude as a function of the dual comb’s power. The bule point show the measurement points for different power levels and the black line shows the linear fitting of the measurement points.

The actual output power of the dual-comb source was adjusted by an erbium-doped fiber amplifier (EDFA) from 28 mW to 100 mW. The normalized signal, plotted as a function of laser power and measured at the highest acoustic beat note (at 900 Hz), is illustrated in Fig. 7. A linear fitting routine was employed for analysis. The excellent fit attests to the linear response of the system to the optical power and affirms that the system did not reach a saturation condition.

### 3.3 SNR with acquisition time

**Fig. 8:**
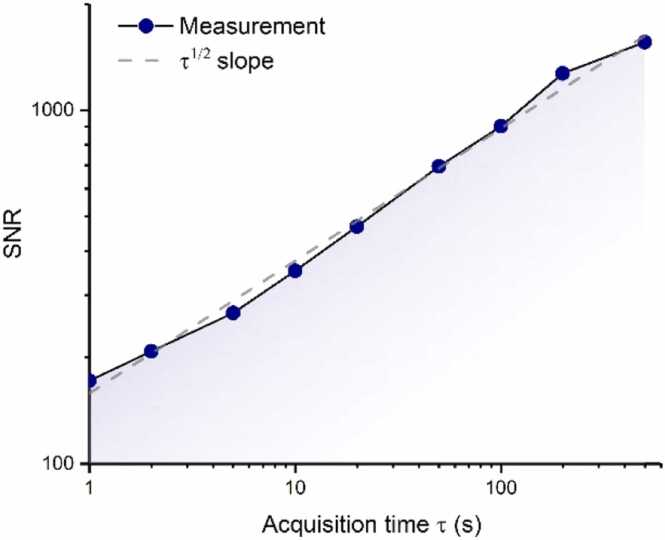
Signal-to-noise (SNR) as a function of the acquisition time $\tau^{\;}$. The SNR for different acquisition time is plotted as blue point, and the black dashed line indicates the ideal case where the SNR increases proportionally to $\tau^{1/2}$.

To examine the impact of extended recording durations (represented by time τ) on the enhancement of the SNR, the non-resonant PAC is filled with N_2_-diluted C_2_H_2_ at a concentration of 500 ppm, probed with a power of 50 mW. The SNR is calculated using the same method as illustrated in Fig. 6. Acquisitions of varying durations undergo processing, and the SNR of the highest acoustic beatnote (at 900 Hz) is assessed relative to the acquisition time τ, spanning acquisition time from 1 s to 500 s. As depicted in Fig. 8, the measurement SNR (blue point) demonstrates an increase with τ^1/2^, aligning well with the τ^1/2^ scaling (depicted by the black dashed line). This outcome implies that extending the acquisition time even further could be exploited to enhance the SNR.

## 4 Conclusions

In conclusion, we have proposed and experimentally demonstrated an innovative approach for a cantilever-enhanced DC-PAS system. This approach combines a high-sensitivity cantilever sensor combined with a non-resonant PAS, which offers highly sensitive, compact size and wavelength-independent for trace gas detection. The adopted FPI-based optical cantilever microphone is specifically designed for sensitive and stable acoustic pressure detection at the low frequency (∼kHz). The non-resonant PAC provides a flatness response (∼kHz), ensuring a uniform acoustic gain for the multiheterodyne sound waves. Furthermore, the multiheterodyne sound waves, generated through the interaction between the dual-comb and the gas molecules, are effectively mapped into the response region of the cantilever microphone. The photoacoustic spectra of C_2_H_2_ have been obtained by Fourier transforming the interferograms generated from the FPI within a short measurement time—specifically, as short as 1 s in this study. Additionally, we have demonstrated the linear response of the cantilever-enhanced DC-PAS system to changes in concentration and power. With a dual-comb power of 100 mW and an average time of 10 seconds, a MDL of 860 ppb was achieved. To further enhance the SNR, an even longer acquisition time can be adopted (as long as 500 s), but a longer acquisition time may not further enhance the signal significantly, because 1/f noise is gradually dominating. This technology opens avenues for exploration in diverse fields, including nonlinear spectroscopy, advanced multi-component gas detection methodologies, and precision molecular spectroscopy techniques.

## CRediT authorship contribution statement

**Hongpeng Wu:** Validation, Methodology, Funding acquisition. **Xiaoli Liu:** Investigation, Formal analysis, Data curation. **Jiapeng Wang:** Writing – original draft, Methodology, Data curation. **Chaofan Feng:** Software, Investigation, Formal analysis. **Ruyue Cui:** Methodology, Formal analysis, Data curation. **Gang Wang:** Software, Investigation, Conceptualization. **Yong Wang:** Software, Project administration, Investigation. **Zhenfeng Gong:** Validation, Resources, Project administration. **Lei Dong:** Writing – review & editing, Visualization, Project administration, Funding acquisition, Conceptualization.

## Declaration of Competing Interest

No potential conflict of interest was reported by the authors.

## Data Availability

Data will be made available on request.
